# Coordinating Mechanisms Are More Important Than Team Processes for Geographically Dispersed Emergency Dispatch and Paramedic Teams

**DOI:** 10.3389/fpsyg.2022.754855

**Published:** 2022-03-08

**Authors:** Bjørn Helge Johnsen, Roar Espevik, Jarle Eid, Øyvind Østerås, Johan Kolstad Jacobsen, Guttorm Brattebø

**Affiliations:** ^1^Department of Psychosocial Science, University of Bergen, Bergen, Norway; ^2^Centre for Crisis Psychology, University of Bergen, Bergen, Norway; ^3^Swedish Defence University, Stockholm, Sweden; ^4^Department of Anaesthesia and Intensive Care, Haukeland University Hospital, Bergen, Norway; ^5^Department of Clinical Medicine, University of Bergen, Bergen, Norway

**Keywords:** medical first responder teams, shared mental models, team processes, coordinating mechanisms, performance

## Abstract

In recent decades there has been an increased emphasis on non-technical skills in medical teams. One promising approach that relates teamwork to medical efficiency is the theory of Shared Mental Models (SMM). The aim of the present study was to investigate the suitability of the Shared Mental Model approach for teamwork between operators in emergency medical communication centers and the first line ambulance personnel in real-life settings. These teams collaborate while working from geographically dispersed positions, which makes them distinct from the kinds of teams examined in most previous research on team effectiveness. A pressing issue is therefore whether current models on co-located teams are valid for medical distributed teams. A total of 240 participants from 80 emergency medical teams participated in the study. A team effectiveness model was proposed based on identified team coordinating mechanisms and the “Big five” team processes. Path analyses showed that SMM was positively associated with team effectiveness (i.e., performance satisfaction and situational awareness) and negatively related to mission complexity. Furthermore, the coordinating mechanisms of SMM and Closed Loop Communication was positively related to “Big five” team scores. However, no effects were found for the “Big five” team processes on effectiveness, which could indicate that the model needs to be adjusted for application to geographically dispersed teams. Possible implications for team training of distributed emergency response teams are discussed.

## Introduction

Emergency medical organization in Norway is centered around emergency medical communication centers (EMCC). The EMCC’s are the primary point of contact in case of a critical medical predicament. The personnel at the EMCC consist of registered nurses or paramedics who have completed mandatory training in emergency communication, emergency medical system knowledge, and the technical skills required for the job. The role of the EMCC is to provide medical advice to the caller based on the Norwegian Index for Emergency Medical Assistance, a decision support tool (Norwegian Medical Association, 2009). This involves conducting triage, dispatching and directing the ambulances, and acting as a communication-hub between the ambulances, general practitioners, and external entities like the local police operational headquarters or fire and rescue units. The EMCC-operator and the ambulance personnel can be considered as constituting a team, since they consist of two or more persons coordinating their activities toward a common goal (Parelta et al., 2018). Given the critical nature of the missions and the need for a rapid response, combined with the complexity of the EMCC operations, there is a constant need to monitor and improve the performance of these frontline services. One promising strategy for augmenting EMCC operations has been to focus on generic and specific non-technical skills (NTS) to improve teamwork. The importance of NTS in health care is documented by Flin and Maran (2004) who outlined a framework for training medical teams. Furthermore, the NTS of team leadership was stressed by the UK General Medical Council (2016), the Academy of Royal Medical Colleges and the UK National Health Service (2010) in their “Medical Leadership Competency Framework.”

A promising development in the study of NTS in medical teams is the so-called Shared Mental Model (SMM) approach (Johnsen et al., 2017). The core of the SMM approach holds the view that effective performance during high workload operations relies upon a shared description, understanding and prediction of the occurring events (i.e., SMM; Weld et al., 2016). The shared cognitive construct generates an immediate and internalized understanding of members’ coordination, decisions, and actions. Based on a literature review covering 20 years of research on team effectiveness, Salas et al. (2005) proposed a model where SMM, Trust, and Closed-Loop Communication (CLC) acted as coordinating mechanisms in order to develop and/or maintain effective team processes. Based on the literature review, they distilled five team processes called “the Big five in teamwork,” which included team leadership, mutual monitoring, team adaptation, team orientation and mutual support behavior. Thus, a relationship between coordinating mechanisms and performance was inferred to be mediated by these five team processes (Salas et al., 2005; Uitdewilligen et al., 2018).

The model proposed by Salas et al. (2005) found empirical support from qualitative and exploratory studies of nursing teams from acute, intensive care, and maternity units (Kalisch et al., 2009). By means of focus groups and probing questions, centered on both coordinating mechanisms as well as team processes, the results indicated that these factors were related to team performance. Another study involving the two coordinating mechanisms of SMM and Trust (McComb et al., 2017) found differences in SMM concerning role responsibilities between physicians and nurses as well as a differential reporting of trust between the two professions. Although the study from McComb et al. (2017) did not test the total SMM model and its relation to team performance, it indicated that there were differences in the use of coordinating mechanisms between nurses and physicians.

In a comparison between two approaches to NTS in health care, Westli et al. (2010) showed that SMM measures explained performance indicators over and beyond the effect of Anesthetists’ NTS Behavioral marker system (Fletcher et al., 2003) in acute medical teams. This was also evident in a study of behavioral markers of SMM in high performance trauma teams where leaders of these teams more frequently displayed behavioral markers of SMM compared to less effective teams (Johnsen et al., 2017). However, both studies investigated co-located teams, where the team processes could be observed by all team members. The EMCCs, on the other hand, are characterized by team members solely dependent on verbal coordination while working from separate locations. Sharing information on status, intentions and resource coordination is done exclusively *via* communication technology. It is therefore reasonable to assume that geographical disparity and the dynamic nature of the situation create a challenge for team processes like Mutual Monitoring, Adaptation and Support Behavior.

Modes and ways of communicating have an influence on SMM, and the introduction of new technology heavily influences the flow of information between team members (Meynard and Gilson, 2014). Historically, there has been a change in how teams execute teamwork, which is driven by advances in information technology. Most studies on the development of SMM and its relations to team effectiveness is anchored in an understanding that the relation is motivated by increased interaction between team members, increased communication, and training (Kraiger and Wenzel, 1997; Mjelde et al., 2015). However, little attention has been given to the effect of using only technological means to communicate on the relationship between coordinating mechanisms, team processes and team effectiveness. Both in general and for operational professionals in particular, communication technology has changed teamwork from face-to-face to more virtual interactions where technological assets play an increasingly important role in the command, control, and coordination of distributed emergency response units. The potential for information exchange with technological aids could facilitate, impair, or have a neutral effect on team effectiveness (Curseu et al., 2008; Algesheimer et al., 2011; Meynard and Gilson, 2014). Teams could vary in the degree of reliance on technological tools to communicate, coordinate and execute team processes. Meynard and Gilson (2014) investigated the development of SMM in teams with unknown team members, the interaction of task interdependence, and technological attributes. However, the focus of that study was the development of a new model and not testing the total model proposed by Salas et al. (2005). To our knowledge, no studies on virtual teams have investigated the total effect of both coordinating mechanisms and team processes on team effectiveness in the same analyses using real-life scenarios. Taken together, there is a need for empirical studies of team processes and performance of geographically distributed team members in the Emergency Medical Services (EMS) domain.

**FIGURE 1:**
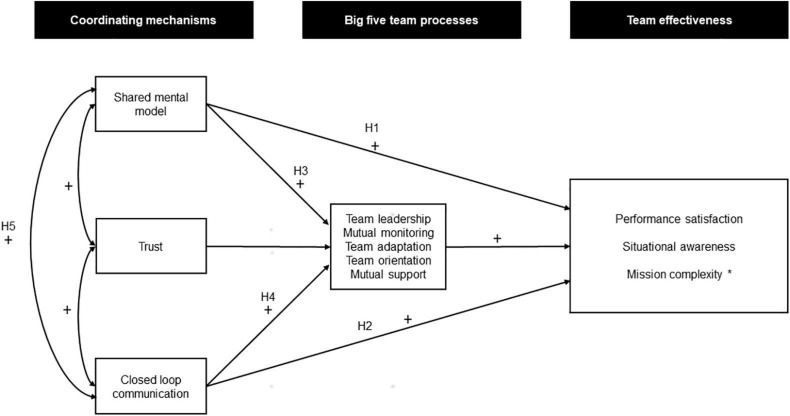
The proposed model of the study. The model is separated for the three coordinating mechanisms of trust, shared mental model and closed loop communication. The Big five team processes are expressed as a composite score of team leadership, mutual monitoring, team adaptation, team orientation and mutual support. Team effectiveness is represented by EMCC operator’s evaluation of performance satisfaction, situational awareness and mission complexity. The predicted directions of the associations are marked on the arrows (*Negative association between team processes and measure of team effectiveness).

The overarching aim of the present study was to test the fitness of the SMM approach on EMS teams where the coordinator is dispersed geographically from the other team members. An evidence-based, theoretical approach (e.g., SMM) could provide more targeted and effective approaches to the education and training of EMS teams. The suitability of the model could be investigated by using path analysis to explain the separate impact on effectiveness ratings of coordinating mechanisms and team processes. The specific hypotheses are outlined as paths (see Figure 1) and listed below:

Hypothesis 1: A direct positive effect of SMM on the measures of performance satisfaction and situational awareness as well as a direct negative effect on perceived mission complexity.

This hypothesis is based on the abundance of studies that has demonstrated an effect of the coordinating mechanism of SMM on team effectiveness (Espevik et al., 2006, 2011a,b; Curseu et al., 2008; Johnson et al., 2011; Meynard and Gilson, 2014; Johnsen et al., 2017).

Hypothesis 2: A direct positive effect of Closed Loop Communication (CLC) on the measures of performance satisfaction and situational awareness as well as a direct negative effect on perceived mission complexity.

CLC has also been shown to affect team output. A link between CLC and performance is found in other domains like firefighter teams (Jouanne et al., 2017). However, in the medical domain, a leadership style involving CLC has been less prominent despite research suggesting that CLC may be vital to success in operational teams (Undre et al., 2006; Härgenstam et al., 2013; Mesmer-Magnus et al., 2017; El-Shafy et al., 2018).

Hypothesis 3: A positive path flowing from SMM through team processes and further on to team effectiveness for the measures of performance satisfaction and situational awareness. Since lower mission complexity represent increased team effectiveness a negative association was hypothesized between team processes and mission complexity in this mediation analyzes.
Hypothesis 4: A positive path flowing from CLC through team processes and further on to team effectiveness for the measures of performance satisfaction and situational awareness. Also, for this mediation analyses lower mission complexity represent increased team effectiveness. Thus, a negative association was hypothesized between team processes and mission complexity.

The pathway from coordinating mechanisms (hypotheses 3 and 4) through team processes is based on Salas et al. (2005), and the assumption that SMM and CLC generate and maintain team processes is well supported in the literature (Johnson-Laird, 1983; Rentsch and Klimoski, 2001; Espevik et al., 2006, 2011b; DeChurch and Mesmer-Magnus, 2010; Johnson et al., 2011; Meynard and Gilson, 2014; Jouanne et al., 2017).

Hypothesis 5: All three coordinating mechanisms, including trust, would be positively inter-correlated and positively correlated with team processes. It could be argued that levels of trust influence coordination within a team, acceptance of mutual monitoring, willingness to support each other, inclination to share information as well as a proclivity to adapt strategies based on input from other team members (McComb et al., 2017).

No specific hypothesis was generated regarding trust and its effect on team output, since trust differs from the other coordinating mechanism by not involving behavior directly related to information sharing in order to alter the cognitive representations of the situation. Instead, trust works more in line with belief systems involving emotional aspects of team membership.

## Materials and Methods

### Participants and Environment

A total of 240 participants constituting 80 teams participated in the study. The teams consisted of two ambulance personnel and one dispatcher in the Bergen EMCC. Bergen is the second largest city in Norway, and Bergen EMCC covers approximately 450,000 people, with about 58,000 ambulance missions a year. Fifty-six percent of the operators reported less than one year of EMCC experience, 40% had between one and 12 years of experience, and 4% reported more than 12 years of experience.

### Measures

SMMs, trust, performance satisfaction, situational awareness, and mission complexity were measured using a five-item, single-page, paper-based, self-report questionnaire. Each construct was measured using a single item 100 mm visual analog scale (VAS; McCormack et al., 1988). The VAS was constructed as a solid line with anchor points representing opposite descriptors (e.g., very low to very high).

Item details are as follows:

*Shared Mental Models.* Shared mental models. SMMs were rated with the question “To what degree did the EMCC-operator and ambulance personnel create a shared understanding/SMM of the mission (team members updated each other on aims, situations, priorities, and internal/external needs)?”
*Trust.* Trust was measured with the item: “To what degree did the EMCC-operator and the ambulance personnel trust each other in order to ensure that the mission was completed in the best possible way? Mark your assessment of trust between the EMCC-operator and ambulance personnel.”
*Performance satisfaction.* Subjective ratings are thought to be better measures of performance than objective measures since objective measures fail to account for the impact of non-controllable third variables (Nicholls et al., 2012). Sports psychology research has advocated for the use of subjective performance measures given the importance of emotion in performance appraisal (i.e., feelings of satisfaction; Nicholls et al., 2012). Therefore, performance satisfaction was measured using the item: “How satisfied were you with the performance of the team?”
*Situational awareness.* Operators’ perception of the team’s overall situational awareness was measured using the item: “To what extent did the EMCC-operator and the ambulance personnel maintain the best possible overview of the situation?”
*Mission complexity.* Mission complexity was measured with the item: “To what extent did you perceive the mission as complex?”

Closed-loop communication and all five team process variables were measured using frequency counts derived by study personnel from audio recordings of the interactions between EMCC operators and ambulance personnel.

*Closed Loop Communication.* Scores on CLC were obtained from verbal statements of the logged communication between the EMCC-operator and the ambulance personnel during the execution of the real-life emergencies. The number of confirmations acted as a measure of the third coordinating mechanism.
*Team processes.* By analyzing the logged authentic communication between the EMCC-operator and the ambulance personnel, frequencies of the “Big five” team behavior described by Salas et al. (2005) were recorded. Table 1 shows the five team processes including a generic description and examples. The frequencies were summed making an aggregated score for team processes.

**TABLE 1:** Big-five team processes based on Salas et al. (2005) including behavioral markers, generic observed behavior and examples.

Team processes	Behavioral markers	Observed behavior	Examples
Team leadership	Coordination of team	Organizing, prioritizing and updating	Will arrive (at the E.R) in … minutes.
Team orientation	Team goals above own goals	Suggestions, recommendations, involvement in tasks	Patient reporting pain in ….region. Checking response and suggest ….
Monitoring	Assess performance	Controlling, checking and self-correction	Do you copy? » and »understood»?
Adaptation	Adjust behavior to environment	Change in plans	It might be best if you….
Support	Predicting needs of team-members and share workload	Taking over tasks, offering resources and information	“Would you like to speak to the local GP?” I will make the necessary arrangements

### Procedure

According to Norwegian regulations, ethical approval for anonymous non-health-related data or quality improvement studies is not required. However, a formal letter of acceptance confirmed the approval of the study by the EMCC-administration, based on regulations in the Health Personnel Act (Helsepersonelloven) §26. Since a prerequisite for conducting the study was that data collection should not intervene with ongoing activity at the EMCC, several meetings were held with key personnel to develop the short questionnaire and entertain mutual understanding. Verbal and written information was presented to the EMCC-operators with a focus on anonymity and consent. Only written information was presented to the ambulance personnel.

As a standard procedure, all communication, internal in the EMCC and external toward the ambulances, are stored in a voice-recorded database. Thus, all voice recordings included in the present study were based on real-life ambulance missions. The authentic voice recordings consist of complete audio recordings from the emergency call to the end of the mission. Since the study was based on real life emergencies, team composition (e.g., EMCC operator and ambulance workers) were done using standard protocol for handling emergency calls in the EMCC. This includes random assignment to team based on availability of EMCC-operator and ambulance, distance to the emergency and urgency of need for care.

By retrieving the logged verbal communication, the behavioral markers of team processes and CLC could be scored. Based on the unique identification number of the mission, the anonymous data from the questionnaires were combined with an anonymized version of the stored communication (i.e., the names of the EMCC and ambulance personnel, and patient, were deleted). The variables were extracted by manually scoring pre-defined categories of verbal statements (see Table 1) from digital sound-files played on a computer. The scoring was done by three research assistants, trained in this specific methodology.

The questionnaire administered to the EMCC-operators was filled in immediately after completion of a mission.

### Analyses

Reliability and correlational analyses were conducted using SPSS 25. Two-tailed Pearson product-moment correlation coefficients were used in the bi-variate correlational analyses, and intra-class correlations were used to test the inter-rater reliability of the scoring of the coordinating mechanisms and the “Big five” team processes. The intra-class correlations were based on 25 voice recordings scored by all raters. Path analyses were conducted using AMOS 25, testing the *a priori* hypothesis model show in Figure 1. Separate analyses were performed for each of the three dependent variables of performance satisfaction, mission complexity and situational awareness. In the proposed model SMM, trust, and CLC acted as exogenous variables, while team processes and dependent variables acted as endogenous variables. The effects are based on generalized least square estimates. Ninety percent confidence intervals (90% CI) were determined using 1,000 bootstrapped samples. The proportion of explained variance in variables was calculated using multiple squared correlations. To compare the fit of the proposed model with the observed data, Comparative Fit Indexes (CFI) were computed for each dependent variable. A value close to 0.95 indicates a good fit between the hypothesized model and the observed data, whereas values in the range of 0.90–0.95 are considered acceptable (Hu and Bentler, 1999).

## Results

### Descriptive Data

In the present study, 92% of the missions were coordinated by EMCC-operators with a background as ambulance paramedics, whereas 6% of the missions were coordinated by operators with a dual background as a registered nurse and ambulance paramedic (2% missing data).

The measure of “Big five” team processes showed an alpha-value of 0.593. This low alpha value indicated the multi-dimensionality of the measure. This is to be expected since the variable is a composite measure of five different team processes. Three raters scored the communications showing an intra-class correlation ranging from *r* = 0.726 to *r* = 0.967 on the different dependent measures. Means and standard deviations are presented in Table 2.

**TABLE 2:** Means (M), standard deviations and inter-correlations for all variables in the proposed model.

	M	SD	1	2	3	4	5	6
Shared Mental Model (1)	78.69	13.86						
Trust (2)	80.94	13.75	0.52**					
Closed Loop Comms. (3)	2.03	1.75	–0.06	–0.10				
Team processes (4)	7.06	7.27	0.04	–0.13	0.79**			
Performance satisfaction (5)	83.06	12.02	0.60**	0.78**	–0.13	–0.09		
Situational awareness (6)	77.24	15.04	0.56**	0.38**	0.08	0.18	0.52**	
Mission complexity	27.71	22.34	−0.46**	−0.43**	0.13	0.11	−0.33**	−0.09

**p < .01.

### Relationship Between the Shared Mental Model Approach and Measures of Effectiveness

Positive correlations were found between SMM and the dependent measures of performance satisfaction and situational awareness (see Table 2). A significant negative correlation was revealed between SMM and complexity of the situation. The same pattern was found for the mechanism of trust. The only significant correlation involving CLC was a positive association to team processes, and the only inter-correlation between the mechanism was a relation between trust and SMM. These results partially support hypothesis 5.

### The Fitness of the Shared Mental Model Approach in Explaining Team Effectiveness

#### Shared Mental Models and the Prediction of Performance Satisfaction

A significant positive direct effect from SMM to Performance satisfaction (*p* < 0.001; see Table 3) was found. Thus, supporting our first hypothesis. No direct effect of CLS on performance satisfaction was found. Thus, hypothesis 2 was not supported. The present analysis also revealed a positive total effect (direct and indirect) on the path from SMM to performance satisfaction, using team processes as a mediator (*p* < 0.002; 90% bootstrapped CI = 0.375 to 0.604). No paths flowing from CLC through team processes were found (not supporting hypothesis 4). The path analysis further showed significant positive paths from SMM and CLC to team processes (*p* < 0.04 and *p* < 0.001, respectively). No effect of team processes on performance satisfaction was revealed. The multiple squared correlation analyses showed that team mechanisms explained 64% (*R*^2^ = 0.64) of the variance in team processes. Sixty-two percent of the variance in performance satisfaction was explained (*R*^2^ = 0.62). When fitting the proposed model onto the observed data, a CFI of 0.573 occurred. This indicates a low fit for the model.

**TABLE 3:** Regression weights for the proposed paths in the model predicting the three dependent variables (DV) of subjective evaluation of performance satisfaction, situational awareness, and mission complexity (complexity).

Paths	Performance satisfaction		Situational awareness		Complexity	
	Un-standardized	β-weights	Un-standardized	β-weights	Un-standardized	β-weights
**Direct effects**						
SMM - > DV	0.523***	0.78	0.599***	0.561	−0.744***	−0.483
SMM - > Team processes	0.146*	0.278	0.087*	0.167	0.09*	0.171
Team Processes - > DV	−0.167	−0.131	0.375	0.185	0.381	0.13
Trust - > Team processes	−0.191	−0.269	−0.077	−0.143	−0.082	−0.148
Closed Loop Comms. - > DV	−0.118	−0.022	−0.261	−0.031	0.049	0.004
Closed Loop Comms - > Team processes	3.186***	0.767	3.247***	0.782	3.244***	0.781
**Indirect effects**						
SMM - > Team processes - > DV	−0.024	−0.036	0.033	0.031	0.034	0.022
Trust - > team processes - > DV	0.032	0.035	−0.029	−0.026	−0.031	−0.019
Closed Loop Comms. - > Team processes - > DV	−0.532	−0.1	1.219	0.144	1.236	0.101
**Total effects**						
SMM - > DV	0.498**	0.743	0.632**	0.592	−0.710**	−0.460
Closed Loop Comms. - > DV	−0.649	−0.122	0.958	0.113	1.286	0.105

*Table presents direct, indirect and total effects. The effects are separated for unstandardized and standardized (β) weights.*

*p < 0.05; **p < 0.01; ***p < 0.001.

#### Shared Mental Models and the Prediction of Situational Awareness

The analysis using situation awareness as an outcome variable revealed a significant positive path from SMM directly to situational awareness (*p* < 0.001; see Table 3), supporting hypotheses 1. No effect involving CLC on situational awareness reached the level of significance. Thus, hypothesis 2 was not supported. The total effect of the path between SMM and situational awareness using team processes as mediator, was significant (*p* = 0.02, 90% bootstrapped confidence interval bounds between 0.456 and 0.800). No significant paths flowing from CLC through team processes on situational awareness was found.

The multiple squared correlation analyses revealed that 35% (*R*^2^ = 0.35) of the variance in situational awareness was explained. Fitting the proposed model on the observed data obtained a CFI of 0.979, indicating a good fit of the model.

#### Shared Mental Models and the Prediction of Mission Complexity

SMM showed a negative direct effect on evaluation of mission complexity (*p* < 0.001; see Table 3), supporting hypothesis 1. No direct effect involving CLC on mission complexity reached the level of significance. Thus, hypothesis 2 was not supported. A total effect of the relation of SMM to complexity, with team processes as a mediator (*p* < 0.002; 90% bootstrapped confidence intervals between −0.951 and −0.457) was also found. No path flowing from CLC through team processes to mission complexity was revealed, providing no support to the fourth hypothesis. No effect of team processes on mission complexity was found. Furthermore, no effects of trust related to any of the measures of team effectiveness were found. The multiple squared correlation for complexity was 0.25, explaining 25% of the variance in mission complexity. An acceptable fit between the proposed model and the data was obtained (CFI = 0.928).

## Discussion

The path analyses showed a direct positive effect of SMM on the EMCC-operator’s evaluation of the team’s execution of the missions (performance satisfaction), the team’s situational awareness, as well as a negative direct effect on perceived mission complexity. Furthermore, both SMM and CLC showed positive predictions of team processes measured as the sum of the “Big five” key team processes. The fit index was shown to vary from good to acceptable for predicting situational awareness and Mission complexity, while the fit for the performance satisfaction model was low.

### Coordinating Mechanisms and Emergency Medical Teams Effectiveness

It is argued that virtual teams (i.e., dispersed team members) operate more autonomously, requiring a higher quality of intra-team teamwork (Johnson et al., 2011). The SMM approach suggests a conceptual model of teamwork characterized by a causal flow from coordinating mechanisms through team processes to performance (Salas et al., 2005). For instance, Mathieu et al. (2000) found that SMM was positively related to team processes and, subsequently through a complete indirect effect, performance in co-located flight simulator teams. However, the prediction of team processes as mediators between mechanisms and performance was not supported in the present study. The results from the path analysis revealed that the EMS-teams’ SMM enhances the evaluation of performance satisfaction and situational awareness, while reducing perceived mission complexity. However, this occurred without the mediating role of the “Big five” team processes. One reason for the findings could be that the transition from face-to-face communication to teamwork in virtual teams actually alters the underlying mechanism, causing successful execution of the team’s mission by increasing the importance of shared cognitions representing vital aspects of the mission. The “Big five” approach describes various processes with behavioral markers that are important for the face-to-face interaction among a co-located team. For a physically dispersed team, there is a need for additional processes to coordinate the team and to establish a shared understanding of task, team and occurring challenges. When team members are separated, the team competencies are reduced, relying more on mechanisms that aims at distributing information equally among team members in order to create sharedness within the team. This need for shared understanding could be fueled by a requirement within the team in order to adapt their behavior to higher order systems. Pre-hospital missions often involve standard medical procedures and a need for integrating these procedures in the medical evacuation chain (i.e., higher order systems). This creates requirements of a shared cognitive representation of the status at the site of the emergency as well as projection in the near future and the requirements are met by the coordinating mechanism of shared mental model. Thus, the need for a shared understanding in order to integrate and coordinate the team’s activity with present and future intentions of the team, guides team interaction based on SMM while verbal markers of team processes are reduced. Our claim is that the increased need for coordinating mechanisms, and especially SMM, could be generalized to other virtual teams. Geographically dispersed team members would perform their professional procedures, but in order to coordinate their activities at present and toward future states, mechanisms that creates sharedness of understanding is crucial. In the absence of visual cues, verbal communication becomes crucial to establish shared mental models.

The main finding of shared mental model being the only predictor of performance lends some support also from studies using a related research approach. Shared mental model is characterized by a shared understanding in the team of tasks, procedures, team members and equipment (Jonker et al., 2011; Sinval et al., 2020). The shared understanding approach shows similar characteristics and refers to the degree to which people concur on the interpretation of significant concepts, when sharing a perspective (mutual agreement) or can act in a coordinated manner (Agredo-Delgado et al., 2020). Agredo-Delgado et al. (2020) investigated the development of shared understanding in teams using computer-supported collaborative work. They found a marked effect on both sharedness within the group as well as performance toward the objectives when focusing on three phases of teamwork. The pre-process phase was composed of activity related to design and specification of objectives and possible disagreements followed by the execution of collaborative activity (process phase). In the post-process phase, a review of the process related to specific objectives was conducted. The design of the Agredo-Delgado et al. (2020) study indicated a mediating role of team processes, Thus, an abundance of empirical data from different research approaches supports the importance of shared cognitive representation of objectives, procedures and team interaction. However, a discrepancy exists of the mediating role of team processes. One reason for the discrepancy could be found in the type of tasks studied. The present study differs from most of the research using the “Big five” approach by studying teams conducting real life critical missions.

Significant aspects when evaluating team performance is the team members’ subjective experiences of mission success. Important factors when rating mission success are the team’s ability to fulfill their objectives when all aspects are considered (i.e., performance satisfaction), gain a correct understanding of the situation (i.e., situational awareness), and obtain clarity regarding the situation (i.e., complexity). SMM being positively related to the evaluation of team situational awareness is also in line with previous research (Macintosh et al., 2009; Saus et al., 2010; Johnsen et al., 2017). Macintosh et al. (2009) found team SA to be related to decision making in face-to-face teamwork in United Kingdom delivery suits. In the present study, this was shown to also be the case for team SA in dispersed medical teams. In a review of teamwork in primary care, Fiscella and McDaniel (2018) emphasized enabling factors for frequent face-to face communication (e.g., space configurations) in order to generate and maintain shared mental models, shared goals, and shared decision making in primary care teams. The negative association between SMM and complexity shows that increased levels of SMM results in a decreased evaluation of the complexity of the situation. This is a notable finding since a perception of reduced situation complexity (e.g., simplicity) may increase teams’ recognition-based decisions (Drury et al., 2012) and prompt the use of standard operating procedures. In a study in the medical domain using the SMM approach, Schraagen (2011) reported complexity as a major risk factor in non-routine pediatric cases. Complexity was related to longer operations as well as more negative patient outcome. However, the study reported that one of the teams studied encountered a more severe case a few weeks after the first, resulting in improved teamwork, higher degrees of attending and employing procedures in a heedful way (Schraagen, 2011). Taken together, the present study expands previous knowledge by showing that SMM is the only team behavior predicting EMS team effectiveness in virtual teams conducting real life missions. Coordinating mechanisms and “The Big-five” team processes.

The results show that the mechanisms of SMM and CLC predict the “Big-five” team processes. This is in line with the predictions of previous models (Salas et al., 2005), where the purpose of the coordinating mechanisms was to support an even distribution of information, fueling the “Big five” team processes resulting in enhanced performance (Uitdewilligen et al., 2018). The effect of team processes on team effectiveness has previously been reported in the medical domain. Studies of team processes within the nursing role (Kalisch et al., 2009), as well as studies of Norwegian trauma teams (Westli et al., 2010; Johnsen et al., 2017), have reported the same predicted effect. However, a common factor in these studies was the co-location of team members involving face-to-face contact. This influence of team process on performance was not supported in our study involving dispersed EMS teams. The direct effect of SMM outperforming an effect mediated by team processes could be caused by an increased need for shared knowledge structures when the team members are located separately.

The present study showed a high correlation between trust and SMM, which is in line with previous findings (Hanna and Richards, 2018). While both SMM and CLC were related to team processes, trust was not. McComb et al. (2017) compared nurses’ and physicians’ level of SMM and trust. They reported a low level of SMM regarding the perceived role responsibilities between nurses and physicians. The two professions showed an equal level of trust toward physicians, while the physicians rated their trust toward nurses lower compared nurses’ evaluation of trust toward their own profession. A seemingly surprising discovery in the present study was the finding of no relation between trust and team processes. Trust could be defined as a belief that the team members will perform expected actions and recognize and protect the interests of their colleagues, as well as a willingness to allow for risk-exposure among members working interdependently (Salas et al., 2005). A possible explanation could be that both CLC and SMM are mechanisms directly involved in generating, maintaining, and altering knowledge structures in the teams, while the impact of trust flows through an indirect emotional component resulting in team members acceptance of being monitored. In addition, for teams consisting of EMCC-operators and paramedics, the function of SMM and CLC are more easily communicated verbally, while signals of trust or lack of trust are harder to communicate and perceive when not being in visual contact. Thus, separating team members geographically alters the mechanisms underlying teamwork and team effectiveness by extinguishing the role of trust. The model fit of the tested model varied from poor (performance satisfaction), to acceptable (mission complexity), to good (situational awareness). This indicates that the model needs to be improved to show more consistency over different indices of team effectiveness.

### Implications

The result from the present study shows the importance of the coordinating mechanism of SMM. Four types of SMMs have been proposed (Cannon-Bowers et al., 1995; Cannon-Bowers and Salas, 1998). This includes shared cognition of equipment, operational task, interaction as well as of the team members. Based on the SMM approach, cross-training is suggested to present an important method to increase shared team knowledge structures (McCann et al., 2000; Marks et al., 2002). Cross training refers to a rotation of team roles following training in one’s own role requirements. This provides an opportunity to experience new learning for all team members and facilitate information sharing that will extend and maintain SMMs. Superior training effects have been found in teams characterized by high levels of SMMs. Espevik et al. (2011b) reported significantly higher levels of performance after only one training session in naval cadet teams exposed to cross-training. Implications of the present study point not only toward the type of training strategy (e.g., cross training), but also toward the training objectives (types of SMM). Thus, in targeting future training it will be paramount to define the type of SMM in need of training (“What”) and the training strategy (“How”) to increase shared cognitions in EMS teams.

### Limitations

Some limitations should be noted. The present study relies on single item measures on several of the variables. From a historical standpoint, reliability and validity issues have discouraged the use of single item measures of psychological constructs. Thus, multiple-item scales are favored (Nunnaly, 1967). However, this view has been challenged and there are several reports supporting the use of single-item variables (Wanous and Reichers, 1996; Wanous et al., 1997). This is founded on empirical data showing high test -retest reliability (Gardner et al., 1998), as well as well as high correlations with multiple item scales (Littman et al., 2006). The validity is also revealed by single item measures effectively predicting outcomes (Littman et al., 2006), including job satisfaction (Wanous et al., 1997; Nagy, 2002). Although there are limitations to single-item measures, potential advantages should also be noted. Advantages like cost-efficiency, greater face validity, and a possible increased willingness of respondents to take time to complete the questionnaire instigated by a less intrusive method compared to the use of multi-item scales.

The item measuring situation awareness is not totally in line with Endsley’s model of situational awareness (Endsley, 1995). The model describes situational awareness as being constituted by three hierarchical organized levels. These levels include detection of critical signals (level 1), understanding of the situation (level 2) and prediction into the near future (level 3). However, the question is meant to capture the core element of the concept by tapping into the individual’s cognitive representation and understanding of the emergency at hand. It could be argued that that this includes level 1 and level 2 in Endsley’s (1995) model.

Both the dependent variables and two of the coordinating mechanisms were evaluated by the EMCC-operators only. This was caused by the study’s intention to not intrude on the ongoing activities. Logistical, methodological, and ethical constraints prevented recordings of data from the ambulance personnel or regarding outcome for the patients. However, the EMCC-operator is a significant team member. By acting as a communication hub, initiating and coordinating the mission, the operators could be viewed as team leaders, and leaders’ evaluation of team effectiveness is imperative.

Another limitation is the lack of control concerning the team members’ identification with the unit studied. The results could be influenced by the participants defining their team membership differently. The ambulance crews could view themselves as part of one team and the EMCC-operators as another team, resulting in measuring inter-team coordination rather than teamwork. Despite this, the units that were studied match the formal definition of teams, justifying the approach of the current study. Finally, nations vary in the organization of their pre-hospital services. Education and training levels, the size of organizations, and the amount and type of operations may differ, limiting the generalizability of the results.

## Conclusion

A consequence of geographically dispersed EMS teams is a change of effective team behavior compared to face-to-face teamwork. The present study reveals a lack of influence with respect to team processes on effectiveness when EMS team members are dispersed. This leads to an increased emphasis on the coordinating mechanism of SMM, demonstrating a direct effect on all the dependent variables of performance satisfaction, situational awareness and mission complexity. The lack of effects on team processes could be due to all team processes being channeled *via* verbal communications only, as well as a need for shared understanding in order to integrated and coordinate the team’s behavior in a higher order system. In this case the evacuation chain from emergency site to higher echelon care. This leaves virtual EMS teams with a high degree of dependency on coordinating mechanisms, for which SMM seem to be crucial. This is important knowledge when determining education and training aimed at increasing the collaboration between EMCC-operators and the ambulance personnel to improve patient safety. However, more research is needed to understand the unique roles of the different types of SMM in predicting performance in different types of teams.

## Data Availability Statement

The raw data supporting the conclusions of this article will be made available by the authors, without undue reservation.

## Ethics Statement

Ethical review and approval was not required for the study on human participants in accordance with the local legislation and institutional requirements. The patients/participants provided their written informed consent to participate in this study.

## Author Contributions

All authors listed have made a substantial, direct, and intellectual contribution to the work and approved it for publication.

## Conflict of Interest

The authors declare that the research was conducted in the absence of any commercial or financial relationships that could be construed as a potential conflict of interest.

## Publisher’s Note

All claims expressed in this article are solely those of the authors and do not necessarily represent those of their affiliated organizations, or those of the publisher, the editors and the reviewers. Any product that may be evaluated in this article, or claim that may be made by its manufacturer, is not guaranteed or endorsed by the publisher.

## References

[B1] Agredo-Delgado V. Ruiz P. H. Mon A. Collazos C. A. Moreira F. Fardoun H. M. (2020). “Validating the shared understanding construction in computer supported collaborative work in a problem-solving activity,” in *Trends and Innovations in Information Systems and Technologies*, eds Rocha A. AdeliLuís H. Reis P. Costanzo S. Orovic I. Moreira F. (Cham: Springer), 203–214. 10.1007/978-3-030-45697-9_20

[B2] Algesheimer R. Dholakia U. M. Gurau C. (2011). Virtual team performance in a highly competitive environment. *Group Organ. Manag.* 36 161–190.

[B3] Cannon-Bowers J. A. Salas E. (1998). *Making Decisions Under Stress.* Washington, DC: APA.

[B4] Cannon-Bowers J. A. Tannenbaum S. I. Salas E. Volpe C. E. (1995). “Defining competencies and establishing team training requirements,” in *Team Effectiveness and Decision Making in Organizations*, eds Guzzo R. A. Salas E., and Associates (San Francisco, CA: Jossey-Bass), 333–380.

[B5] Curseu P. L. Schalk R. Wessel I. (2008). How do virtual teams process information? A literature review and implications for management. *J. Manag. Psychol.* 23 628–652.

[B6] DeChurch L. A. Mesmer-Magnus J. R. (2010). The cognitive underpinnings of effective teamwork: a meta-analysis. *J. Appl. Psychol.* 95 32–53. 10.1037/a0017328 20085405

[B7] Drury M. Conboy K. Power K. (2012). Obstacles to decision making in Agile software development teams. *J. Syst. Softw.* 85 1239–1254. 10.1016/j.jss.2012.01.058

[B8] El-Shafy I. A. Delgado J. Akerman M. Bullaro F. Christopherson N. A. Prince J. M. (2018). Closed-loop communication improves task completion in pediatric trauma resuscitation. *J. Surg. Educ.* 75 58–64. 10.1016/j.jsurg.2017.06.025 28780315

[B9] Endsley M. R. (1995). Toward a theory of situation awareness in dynamic systems. *Hum. Fact.* 37 32–64. 10.1518/001872095779049543

[B10] Espevik R. Johnsen B. H. Eid J. (2011a). Communication and performance in co-located and distributed teams: an issue of shared mental models of team members? *Milit. Psychol.* 23 616–638. 10.1080/08995605.2011.616792

[B11] Espevik R. Johnsen B. H. Eid J. (2011b). Outcomes of shared mental models of team members in cross training and high intensity situations. *J. Cogn. Eng. Dec. Mak.* 5 352–377. 10.1177/1555343411424695

[B12] Espevik R. Johnsen B. H. Eid J. Thayer J. (2006). Shared mental models and operational effectiveness: effects on performance and team processes in a submarine attack team. *Milit. Psychol.* 18 23–36.

[B13] Fiscella K. McDaniel S. H. (2018). The complexity, diversity, and science of primary care teams. *Am. Psychol.* 73 451–467. 10.1037/amp0000244 29792460

[B14] Fletcher G. Flin R. McGeorge P. Galvin R. Maran N. Patey R. (2003). Anesthetists’ Non-Technical Skills (ANTS): evaluation of a behavioural marker system. *Br. J. Anaesth.* 90 580–588. 12697584 10.1093/bja/aeg112

[B15] Flin R. Maran N. (2004). Identifying and training non-technical skills for teams in acute medicine. *Q. Saf. Health Care* 13 80–94. 10.1136/qshc.2004.009993 PMC1765790 15465960

[B16] Gardner D. G. Cummings L. L. Dunham R. B. Pierce J. L. (1998). Single-item versus multiple-item measurement scales: an empirical comparison. *Educ. Psychol. Meas.* 58 898–915. 10.1177/0013164498058006003

[B17] General Medical Council (2016). *Tomorrow’s Doctors: Outcomes and Standards for Undergraduate Medical Education.* Available online at: www.gmc-uk.org/Tomorrow_s_Doctors_1214.pdf_48905759.pdf (accessed December 16, 2016).

[B18] Hanna N. Richards D. (2018). The impact of multimodal communication on a shared mental model, trust, and commitment in human–intelligent virtual agent teams. *Multimod. Technol. Interact.* 2 1–27. 10.3390/mti2030048

[B19] Härgenstam M. Lindkvist M. Brulig C. Jacobsson M. Hultin M. (2013). Communication in interdisciplinary teams: exploring closed-loop communication during in situ trauma team training. *BMJ Open* 3:e003525. 10.1136/bmjopen-2013003525 PMC3808778 24148213

[B20] Hu L. Bentler P. M. (1999). Cutoff criteria for fit indexes in covariance structure analysis: conventional criteria versus new alternatives. *Struct. Equ. Model.* 6 1–55. 10.1080/10705519909540118

[B21] Johnsen B. H. Westli H. K. Espevik R. Wisborg T. Brattebø G. (2017). High performance trauma teams: frequency of behavioral markers of shared mental model displayed by team leaders and quality of performance. *Scand. J. Trauma Resusc. Emerg. Med.* 25:109. 10.1186/s13049-017-0452-3 29126452 PMC5681813

[B22] Johnson T. E. Top E. Yukselturk E. (2011). Team shared mental model as a contributing factor to team performance and students’ course satisfaction in blended courses. *Comput. Hum. Behav.* 27 2330–2338. 10.1016/j.chb.2011.07.012

[B23] Johnson-Laird P. (1983). *Mental Models.* Cambridge, MA: Harvard University Press.

[B24] Jonker C. M. van Riemsdijk M. B. Vermeulen B. (2011). “Shared mental models,” in *Coordination, Organizations, Institutions, and Norms in Agent Systems VI. COIN 2010. Lecture Notes in Computer Science*, vol 6541, eds De Vos M. Fornara N. Pitt J. V. Vouros G. Springer, Berlin, Heidelberg. 10.1007/978-3-642-21268-0_8

[B25] Jouanne E. Charron C. Chauvin C. Morel G. (2017). Correlates of team effectiveness: an exploratory study of firefighter’s operations during emergency situations. *Appl. Ergon.* 61 69–77. 10.1016/j.apergo.2017.01.005 28237021

[B26] Kalisch B. J. Weaver S. J. Salas E. (2009). What does nursing teamwork look like? a qualitative study. *J. Nurs. Care Q.* 24 298–307. 10.1097/NCQ.0b013e3181a001c0 19755879

[B27] Kraiger K. Wenzel L. H. (1997). “Conceptual development and empirical evaluation of measures of shared mental models as indicators of team effectiveness,” in *Team Performance Assessment and Measurement: Theory, Methods and Applications*, eds Brannick M. T. Salas E. Prince C. (Mahwah, NJ: Lawrence Erlbaum), 63–84.

[B28] Littman A. J. White E. Satia J. A. Bowen D. J. Kristal A. R. (2006). Reliability and validity of two single-item measures of psychosocial stress. *Epidemiology* 17 398–403. 10.1097/01.ede.0000219721.89552.51 16641618

[B29] Macintosh N. Berridge E. J. Freeth D. (2009). Supporting structures for team situational awareness and decision making: insights from four delivery suits. *J. Eval. Clin. Pract.* 15 46–54. 10.1111/j.1365-2753.2008.00953.x 19239581

[B30] Marks M. A. Sabella M. J. Burke C. S. Zaccaro S. J. (2002). The impact of cross-training on team effectiveness. *J. Appl. Psychol.* 87 3–13. 10.1037/0021-9010.87.1.3 11916213

[B31] Mathieu J. E. Heffner T. S. Goodwin G. F. Salas E. Cannon-Bowers J. A. (2000). The influence of shared mental models on team process and performance. *J. Appl. Psychol.* 85 273–283. 10.1037/0021-9010.85.2.273 10783543

[B32] McCann C. Baranski J. V. Thompson M. M. Pigeau R. A. (2000). On the utility of experimental cross-training for decision making under time stress. *Ergonomics* 43 1095–1110. 10975175 10.1080/00140130050084897

[B33] McComb S. A. Lemaster M. Henneman E. A. Hinchey K. T. (2017). An evaluation of shared mental models and mutual trust on general medical units: implications for collaboration teamwork and patient safety. *J. Patient Saf.* 13 237–242. 10.1097/PTS.0000000000000151 25706910

[B34] McCormack H. M. David J. D. L. Sheather S. (1988). Clinical applications of visual analogue scales: a critical review. *Psychol. Med.* 18 1007–1019. 10.1017/s0033291700009934 3078045

[B35] Mesmer-Magnus J. Niler A. A. Plummer G. Larson L. E. DeChurch L. A. (2017). The cognitive underpinnings of effective teamwork: a continuation. *Career Dev. Intern.* 22 507–519. 10.1108/cdi-08-2017-0140

[B36] Meynard M. T. Gilson L. L. (2014). The role of shared mental model development in understanding virtual team effectiveness. *Group Organ. Manag.* 39 3–32.

[B37] Mjelde F. V. Smith K. Lunde P. Espevik R. (2015). Military teams – a demand for resilience. *Work* 54 283–294. 10.3233/WOR-162298 27259180

[B38] Nagy M. (2002). Using a single-item approach to measure facet job satisfaction. *J. Occup. Organ. Psychol.* 75 77–86. 10.1348/096317902167658

[B39] Nicholls A. R. Polman R. C. J. Levy A. R. (2012). A path-analysis of stress appraisals. Emotions, coping, and performance satisfaction among athletes. *Psychol. Sport Exerc.* 13:263e270. 10.1016/j.psychsport.2011.12.003

[B40] Norwegian Medical Association (2009). *Norsk Indeks for Medisinsk Nødhjelp (Norwegian Index for Medical Emergency Assistance)*, 3rd Edn, Stavanger: Laerdal Medical A/S.

[B41] Nunnaly J. C. (1967). *Psychometric Theory.* New York, NY: McGraw-Hill.

[B42] Parelta C. F. Lourenco P. R. Lopes P. N. Baptista C. Pais P. N. (2018). Team development: definition, measurement and relationships with team effectiveness. *Hum. Perform.* 31 97–124. 10.1080/08959285.2018.1455685

[B43] Rentsch J. R. Klimoski R. (2001). Why do “great minds” think alike? Antecedents of team member schema agreement. *J. Organ. Behav.* 22 107–120. 10.1002/job.81

[B44] Salas E. Sims D. E. Burke C. S. (2005). Is there a “Big Five” in teamwork. *Small Group Res.* 36 555–599. 10.1177/1046496405277134

[B45] Saus E. R. Espevik R. Eid J. (2010). “Situational awareness and shared mental models: implications for training in security operations,” in *Enhancing Human Performance in Security Operations: International and Law Enforcement Perspectives*, eds Bartone P. T. Johnsen B. H. Eid J. Violanti J. M. Laberg J. C. (Springfield, IL: Charles C. Thomas, Publishers), 143–161.

[B46] Schraagen J. M. (2011). Dealing with unforeseen complexity in the OR: the role of heedful interrelating in medical teams. *Theoret. Issues Ergon. Sci.* 12 256–272. 10.1080/1464536X.2011.564481

[B47] Sinval J. João M. Marques S. C. Uitdewilligen S. Travis M. M. Margarida P. A. (2020). Development of the Referee Shared Mental Models Measure (RSMMM). *Front. Psychol.* 11:550271. 10.3389/fpsyg.2020.550271 33192798 PMC7641634

[B48] Uitdewilligen S. Rico R. Waller M. J. (2018). Fluid and stable: dynamics of team action patterns and adaptive outcomes. *J. Organ. Behav.* 39 1113–1128. 10.1002/job.2267

[B49] UK National Health Service (2010). *Institute for Innovation and Improvement. Medical Leadership Competency Framework; Enhancing Engagement in Medical Leadership, July; Third Edition.* Available online at: https://www.leadershipacademy.nhs.uk/wp-content/uploads/2012/11/NHSLeadership-Leadership-Framework-Medical-Leadership-Competency-Framework-3rd-ed.pdf (accessed December 16, 2016).

[B50] Undre S. Sevdalis N. Healey A. N. Darzi S. Vincent C. A. (2006). Teamwork in the operating theatre: cohesion or confusion?. *J. Eval. Clin. Pract.* 12 182–189. 10.1111/j.1365-2753.2006.00614.x 16579827

[B51] Wanous J. P. Reichers A. E. (1996). Estimating the reliability of a single-item measure. *Psychol. Rep.* 78 631–634.

[B52] Wanous J. P. Reichers A. E. Hudy M. J. (1997). Overall job satisfaction: how good are single-item measures?. *J. Appl. Psychol.* 82 247–252. 10.1037/0021-9010.82.2.247 9109282

[B53] Weld L. R. Stringer M. T. Ebertowski J. S. Baumgartner T. S. Kasprenski M. C. Kelley J. C. (2016). TeamSTEPPS improves operating room efficiency and patient safety. *Am. J. Med. Q.* 31 408–414. 10.1177/1062860615583671 25888549

[B54] Westli H. K. Johnsen B. H. Eid J. Rasten I. Brattebø G. (2010). Teamwork skills shared mental models, and performance in simulated trauma teams. *Scand. J. Trauma Resusc. Emerg. Med.* 18 18–47. 10.1186/1757-7241-18-47 20807420 PMC2939527

